# MIMO Antenna System for Modern 5G Handheld Devices with Healthcare and High Rate Delivery

**DOI:** 10.3390/s21217415

**Published:** 2021-11-08

**Authors:** Saad Hassan Kiani, Ahsan Altaf, Muhammad Rizwan Anjum, Sharjeel Afridi, Zulfiqar Ali Arain, Sadia Anwar, Salahuddin Khan, Mohammad Alibakhshikenari, Ali Lalbakhsh, Muhammad Abbas Khan, Raed A. Abd-Alhameed, Ernesto Limiti

**Affiliations:** 1 Electrical Engineering Department, City University of Science and Information Technology, Peshawar 25000, Pakistan; iam.kiani91@gmail.com; 2 INZA Research Laboratory for Electromagnetic and Microwave Engineering, Multan 60600, Pakistan; draaltaf@gmail.com; 3 Department of Electronic Engineering, The Islamia University of Bahawalpur, Bahawalpur 63100, Pakistan; engr.muhammadrizwan@gmail.com; 4 Department of Electrical Engineering, Sukkur IBA University, Sukkur 65200, Pakistan; Sharjeel.afridi47@yahoo.com; 5 Department of Telecommunication Engineering, Mehran University of Engineering and Technology, Jomshoro 76062, Pakistan; zulfiqar.arain@faculty.muet.edu.pk; 6 State Key Laboratory of Networking and Switching Technology, Beijing University of Posts and Telecommunication, Beijing 100876, China; 7 Department of Business Development and Technology, Aarhus University, 8000 Aarhus, Denmark; sadia@btech.au.dk; 8 Electrical Engineering Department, College of Engineering, King Saud University, Riyadh 11421, Saudi Arabia; khanheu@gmail.com; 9 Department of Signal Theory and Communications, Universidad Carlos III de Madrid, 28911 Leganés, Spain; 10 School of Engineering, Macquarie University, Macquarie Park, NSW 2109, Australia; ali.lalbakhsh@mq.edu.au; 11 Department of Electrical Engineering, Balochistan University of Information Technology, Engineering and Management Sciences, Quetta 87300, Pakistan; Muhammad.abbas@buitms.edu.pk; 12 Faculty of Engineering and Informatics, University of Bradford, Bradford BD7 1DP, UK; r.a.a.abd@bradford.ac.uk; 13 Information and Communication Engineering Department, College of Science and Technology, Basrah University, Basra 61004, Iraq; 14 Electronic Engineering Department, University of Rome “Tor Vergata”, Vial Del Politecnico 1, 00133 Rome, Italy; limiti@ing.uniroma2.it

**Keywords:** MIMO antenna systems, 5G, high gain, internet of things (IOT), wide bandwidth, healthcare, high isolation, high rate delivery

## Abstract

In this work, a new prototype of the eight-element MIMO antenna system for 5G communications, internet of things, and networks has been proposed. This system is based on an H-shaped monopole antenna system that offers 200 MHz bandwidth ranges between 3.4–3.6 GHz, and the isolation between any two elements is well below −12 dB without using any decoupling structure. The proposed system is designed on a commercially available 0.8 mm-thick FR4 substrate. One side of the chassis is used to place the radiating elements, while the copper from the other side is being removed to avoid short-circuiting with other components and devices. This also enables space for other systems, sub-systems, and components. A prototype is fabricated and excellent agreement is observed between the experimental and the computed results. It was found that ECC is 0.2 for any two radiating elements which is consistent with the desirable standards, and channel capacity is 38 bps/Hz which is 2.9 times higher than 4 × 4 MIMO configuration. In addition, single hand mode and dual hand mode analysis are conducted to understand the operation of the system under such operations and to identify losses and/or changes in the key performance parameters. Based on the results, the proposed antenna system will find its applications in modern 5G handheld devices and internet of things with healthcare and high rate delivery. Besides that, its design simplicity will make it applicable for mass production to be used in industrial demands.

## 1. Introduction

Due to the advent of fifth-generation (5G) technology, scientists have been focusing on advanced antenna systems for modern portable devices, such as smartphones, tablets, etc. [1]. This is because 5G technology offers high channel capacity and channel aggregation with low latency over MIMO fading environments [2]. On the other hand, modern portable devices are becoming slimmer and light-weight, and must pose high processing capabilities [3]. To address these aforementioned characteristics, antenna engineers have proposed multiple antennas systems such as MIMO antenna systems. Several studies and analysis were conducted to understand this state-of-the-art concept at the fundamental level [1,2,3]. 

Currently, 4-element based MIMO antenna systems are considered as a standard to obtain high data rates for fourth-generation (4G) and 4G Long-term Evolution (LTE) technologies. Moreover, they are also widely used in the current cellular technology [4,5,6]. In [5], a four element MIMO antenna system in a box-folded shape is proposed for LTE mobile handsets. To improve isolation between the elements, ground slots and L-branches were used as a decoupling structure. This design increases complexity and limits the array prospects for future technologies, such as 5G and their applications in modern devices, for instance tablets and mobile phones. In a similar study, Choi et al. proposed a four element reconfigurable coupled loop antenna system for LTE technology [6]. In [7], Gao et al. presented a four-element based MIMO antenna system packaged in a metallic case of a handset. This system consists of symmetric back-to-back multi-branch monopoles with overall dimension of 80 × 65 × 0.8 mm^3^ that covers the LTE band 42 with isolation level of greater than 25 dB. However, the size of each element within the array is around 15 mm with a long microstrip line that limits the possible extension for 5G MIMO assembly.

It is a well known fact that to achieve high processing capacity with higher multiplexing and spatial diversity characteristics, a higher number of antenna elements (six and above) are required. In other words, six or more antennas may be used in an antenna system to achieve high bandwidth and data rate in 5G technology. Currently, LTE band 42 (2.6 GHz) and band 43 (3.5 GHz) have been set as preferred 5G bands by cellular services. Therefore, several studies and analysis have been proposed for various different MIMO designs as potential 5G candidates for mobile terminals [8,9,10,11,12,13,14,15,16,17,18]. In a study, a six-element unit slot antenna array covering the standard allocated 5G band having dimensions of 136 mm × 68 mm on a 1.6 mm thick FR4 board is presented [19]. In this work, the elements were excited through an L-shaped probe, enabling a channel capacity of 31 bps/Hz for an eight-element MIMO array. In another study [20], an individual element having size of 20 × 1.5 mm^2^ covering LTE band 42 is presented. The elements are placed at the corner of the chassis and no allocation was reserved for 4G technology. Similarly, a multi-element MIMO array in [21] covers the two 5G bands (LTE42/43) in hybrid assembly with four elements printed on the edges of the chassis and on the four corners with ECC less than 0.3 among any two radiating elements. However, such hybrid structure limits the practical application due to design complexity. The purpose of these studies (discussed above) is to provide different designs, assemblies, and chassis for antenna systems of future mobile handsets. An H shape dielectric resonator antenna is presented in [22] or wideband applications. The antenna is simple in structure but it limits its use in chassis application due to its size.

The main purpose and the motivation behind this work is to propose an antenna system which can fulfil the various requirements of 5G technology, such as high bandwidth, high data rate, and low latency. To address these attributes the proposed MIMO system contributions are as follows.

- We have designed an eight-element MIMO antenna system with a simple monopole radiating structure, which can cover sub-6 GHz (LTE band 43) frequency band for 5G technology.
- The isolation between the radiating elements is achieved low without using any decoupling structure and/or technique, and allows space for other RF components and devices.
- In addition, this system can also be easily fabricated and integratable with other RF systems, subsystems, and components. Furthermore, it was ensured that the proposed work must use intra-band contiguous carrier aggregation to increase the data throughput. Next, antenna design of the proposed work is presented in detail.

## 2. Antenna Design

**Figure 1:**
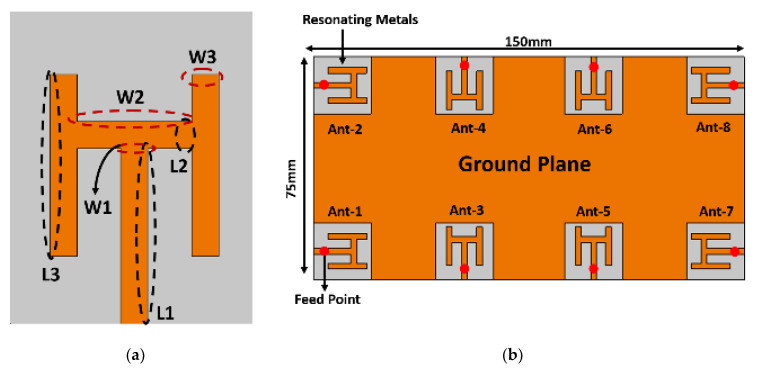
Proposed MIMO antenna system (**a**) Single element. (**b**) Eight-element antenna array.

In this section, a single radiating element, MIMO antenna array, and the working principle of the system is explained in detail. This work presents an H-shaped monopole antenna element, which is designed based on an inexpensive, commercially easily available, and easy to fabricate FR4 substrate. The dielectric constant and the loss tangent of the substrate are 4.4 and 0.02, respectively. The radiating element, feed, and the ground plane, are all kept on the same side of the board, while the copper from the other side of the board is etched to avoid possible short-circuiting of the chassis and allowing space for other RF components and devices. The proposed system is designed on a single double-sided 150 × 75 × 0.8 mm^3^ printed circuit board. A single element is shown in Figure 1a, where the dimensions of the proposed radiating element are as follows: L1 = 13 mm, L2 = 2 mm, L3 = 18 mm, W1 = 2 mm, W2 = 8.5 mm, and W3 = 2 mm. It is worth mentioning that the proposed element is designed according to the commercialized standards of a modern smartphone.

Therefore, it is reasonable to say that the proposed work is consistent with the antenna systems available in the latest commercially available mobile phones.

To arrange the radiating elements in an array, four-elements are etched at the corner of the chassis, while other four-elements are placed within two corner elements on either side of the board. Each antenna element is sharing the same ground plane and a separate 50 ohm feed line, as shown in Figure 1b. It is worthy to mention that these antenna elements are fed in the middle using a coaxial feed. One can choose to feed at any other point until it is half wavelength away at that resonating frequency, but in this work we have chosen the middle point to not only increase the path of the current but also distribute it to all the sides of the radiating element.

**Figure 2:**
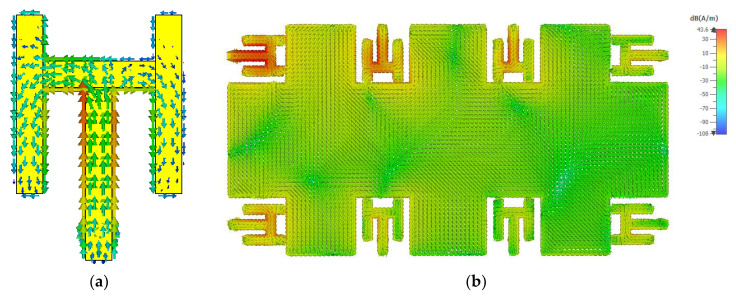
(**a**) Surface current distribution at 3.5 GHz. (**b**) Surface current distribution of the antenna array at 3.5 GHz, when antenna-2 is excited.

It is observed that for the proposed shape, it is resonating at 3.5 GHz with 300 MHz impedance bandwidth of 6 dB. To further analyze the working behaviour of the structure, a surface current density of the antenna at 3.5 GHz is presented in Figure 2a. The surface currents are almost evenly distributed within the structure, specifically, the currents are more focused around the bends and the corners. In addition, the surface current distribution of the antenna array at 3.5 GHz is illustrated in Figure 2b. Here, antenna-1 is excited while other antennas are matched terminated. Please note that, currents are induced on other elements due to mutual coupling between them. Based on the results discussed previously, a detailed analysis has been done to understand the effects of width and the length of different stubs. 

## 3. Results and Discussion

### 3.1. Parametric Analysis

**Figure 3:**
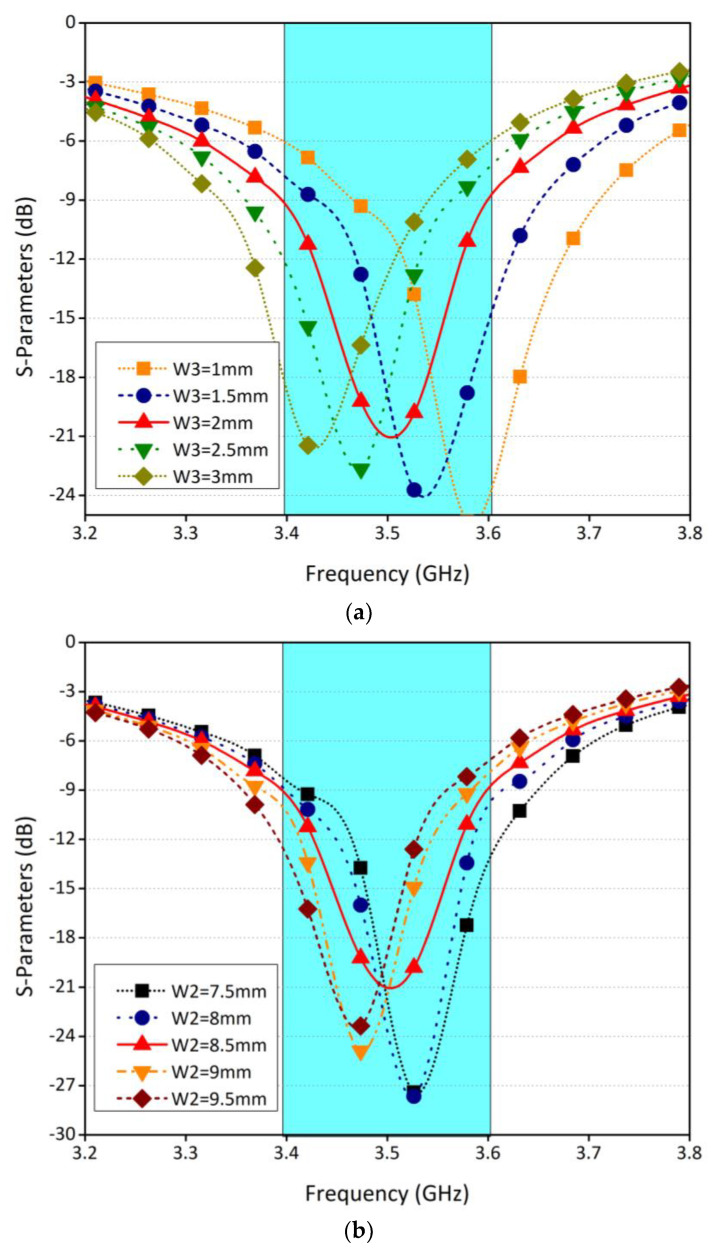

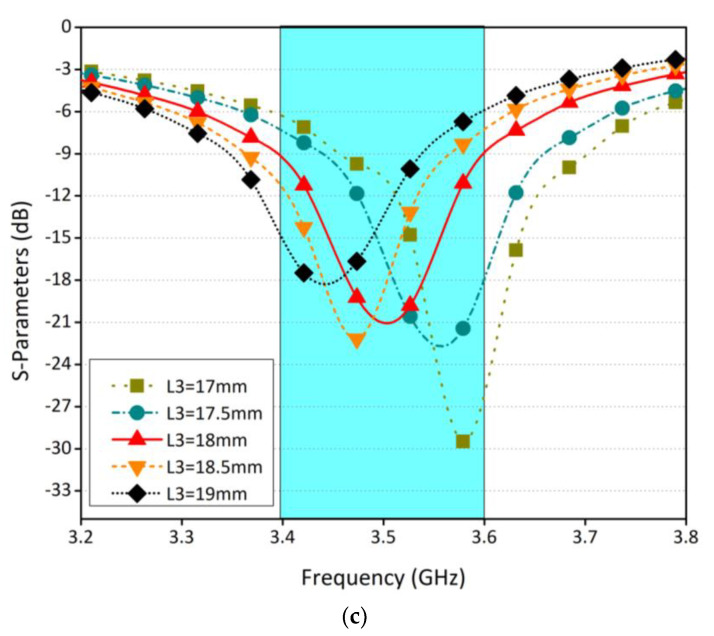
Parametric analysis. (**a**) Reflection coefficient for various values of width W2. (**b**)Reflection coefficient for various values of length L3. (**c**) Reflection coefficient for various values of width W3.

Figure 3a shows the effect of width W2 on the return loss of the antenna. The width is varied from 7.5 mm to 9.5 mm, with an increase of 0.5 mm. It was observed that as we increase the width, the frequency shifts to the right. This is because the length of the antenna was increasing, as a result the frequency was shifting towards the lower side of the band. In another study, the width (W3) and length (L3) are varied, as shown in Figure 3b,c. Both parameters are studied for five different values. Similar conclusions can be drawn for this study and based on these parametric studies, optimum values for different design parameters are selected.

### 3.2. Fabrication and Measurement

**Figure 4:**
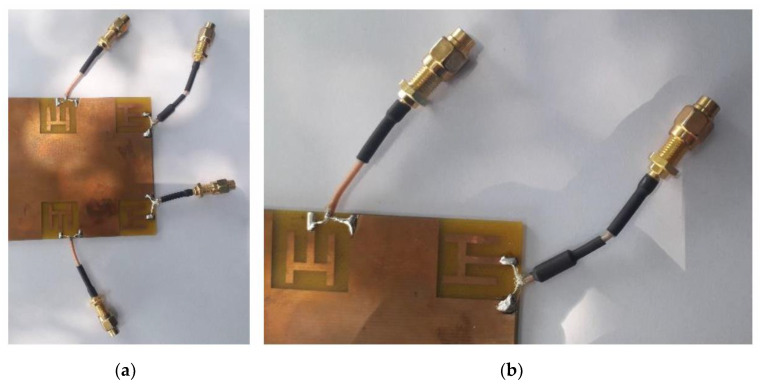

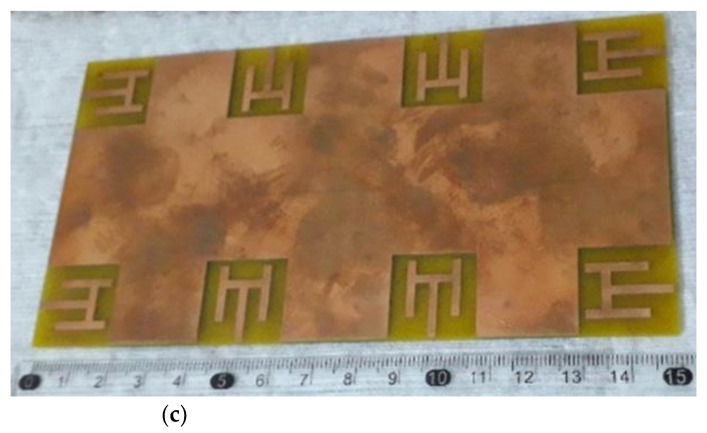
Fabricated Prototype (**a**) View 1 (**b**) Close view 2 (**c**) Full board.

This work is designed in a full-wave electromagnetic software Computer Simulation Technology (CST). The simulated model is fabricated using LPKF D104 PCB milling machine, and the prototype is measured using R\&SZNA67 vector network analyzer. The fabricated model is depicted in Figure 4. Next, different key performance parameters of the system are discussed.

#### 3.2.1. S-Parameters

**Figure 5:**
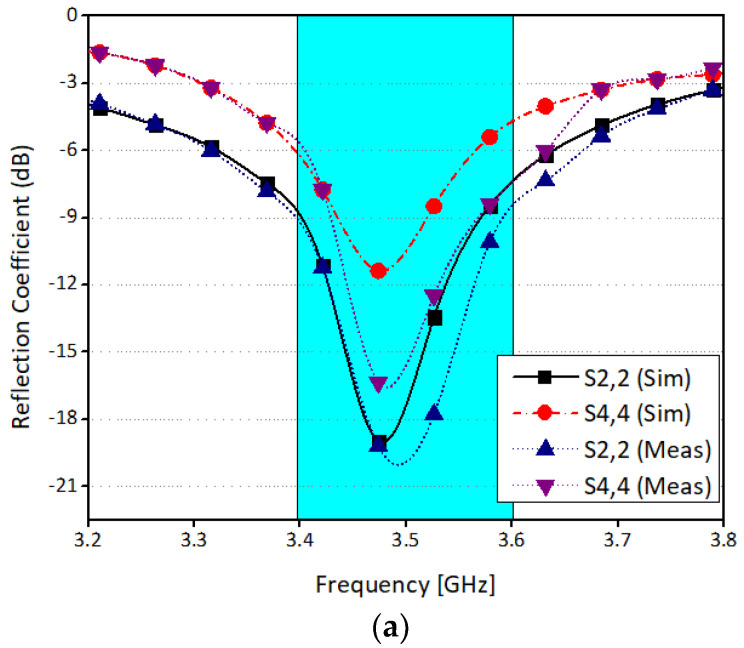

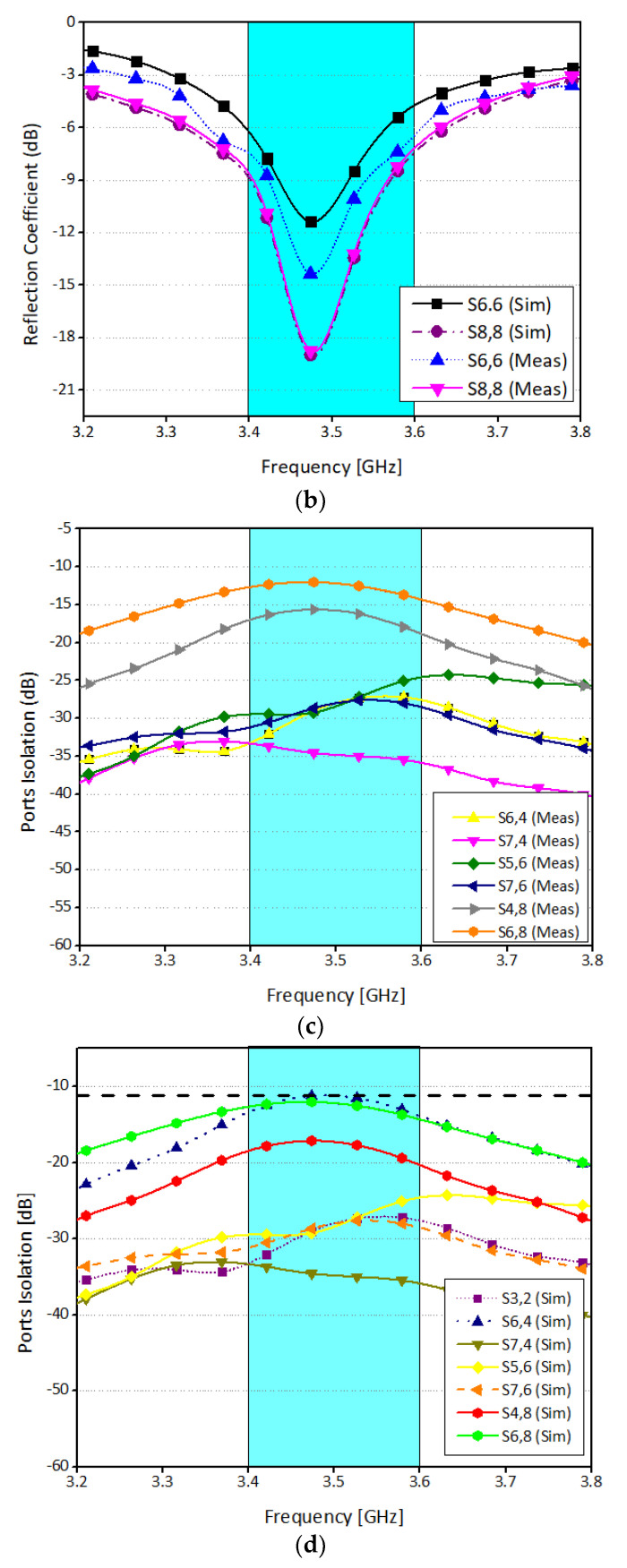
Reflection coefficient of proposed MIMO antenna (**a**) Antenna 2 and 4 Return loss (**b**) Antenna 6 and 8 Return loss (**c**) Ports isolation simulated (**d**) Ports isolation measured.

The scattering parameters of the proposed antenna system are discussed in this section. The elements are arranged in such a way that each side has four radiating elements. For simplicity, radiating elements at one-side of the board are considered. Figure 5a shows simulated return loss of four different ports. It is worthy to mention that the system is resonating at 3.5 GHz with 200 MHz (3.4 GHz to 3.6 GHz) impedance bandwidth of 6 dB. Similarly, Figure 5b illustrates the measured return loss for the same ports. It is found that the computed and the experimental results are in good agreement. A slight variation in results is due to fabrication tolerances and machining accuracy. Figure 5c,d show the simulated and measured isolation between different radiating elements. From the Figures, it can be concluded that the minimum isolation between any two elements is well below −12 dB. The isolation between antennas 6 and 7 are not shown in the above figures, and between antennas 4 and 8 are around −25 dB and not 12 dB. This is because the antennas which are in a close proximity have prominent coupling due to near-field radiations and current flow on the system ground, while for the others the coupling is mainly due to near-field radiations. 

#### 3.2.2. Radiation Patterns

**Figure 6:**
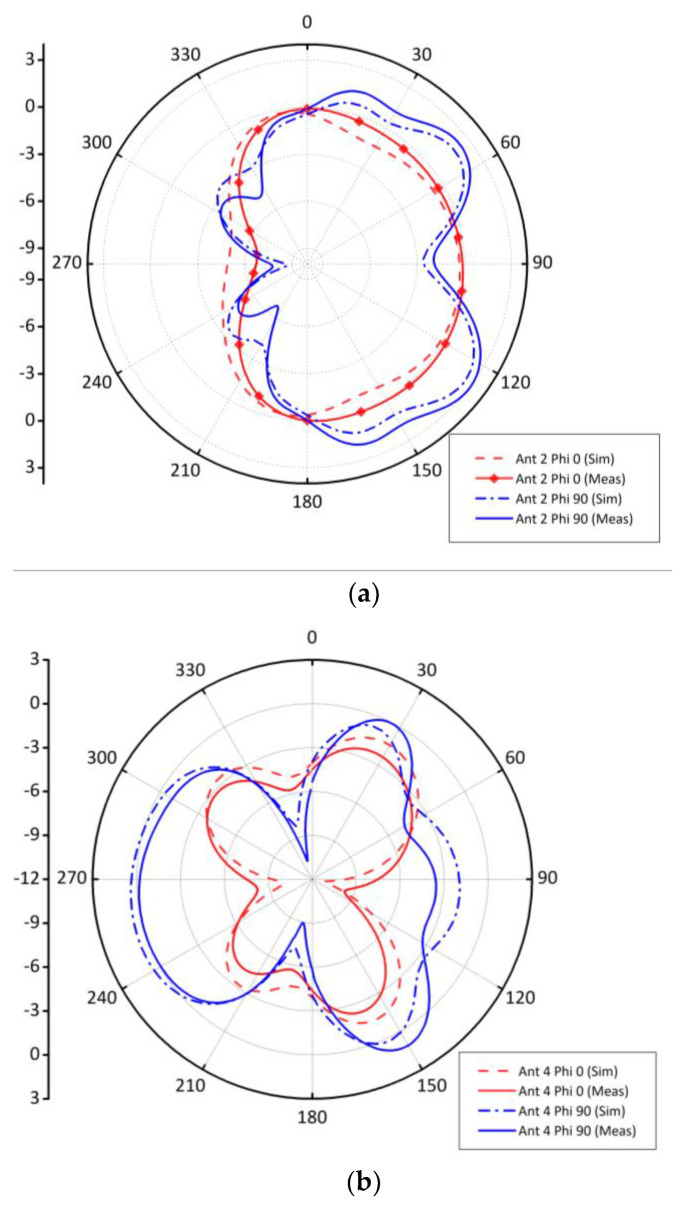

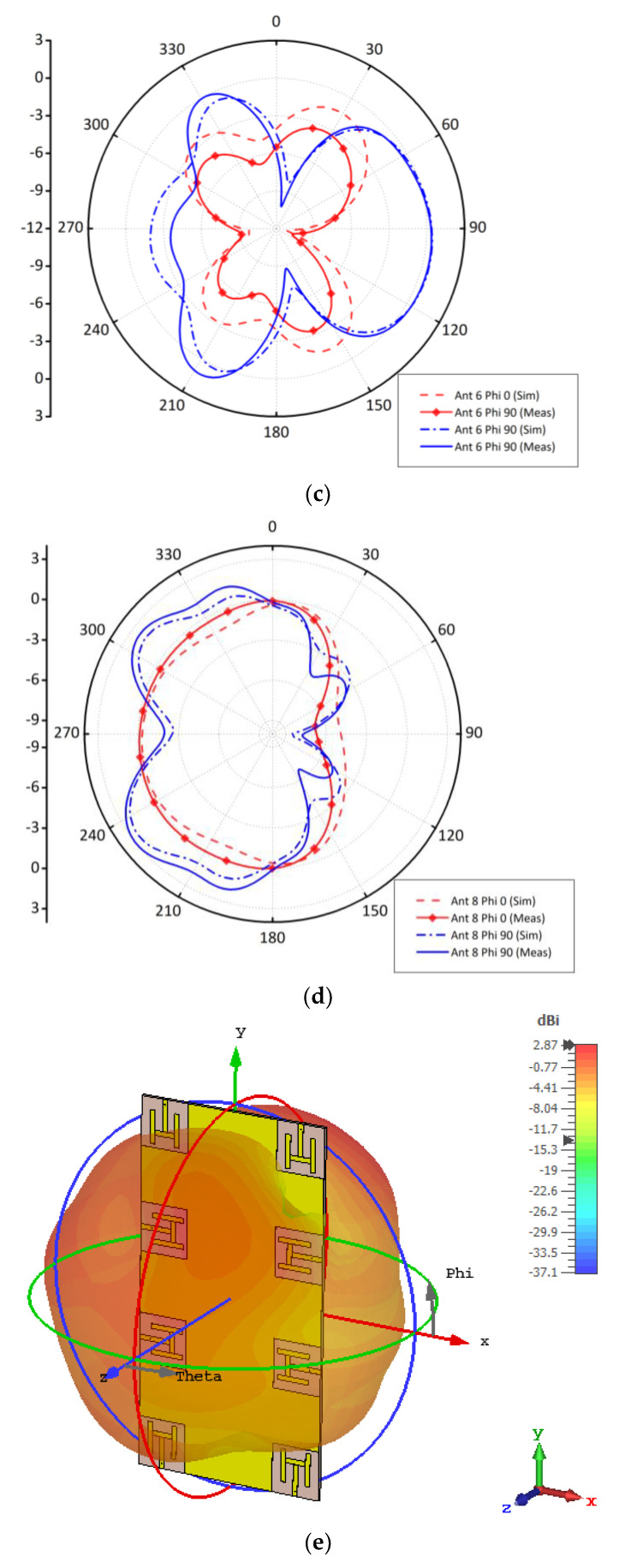

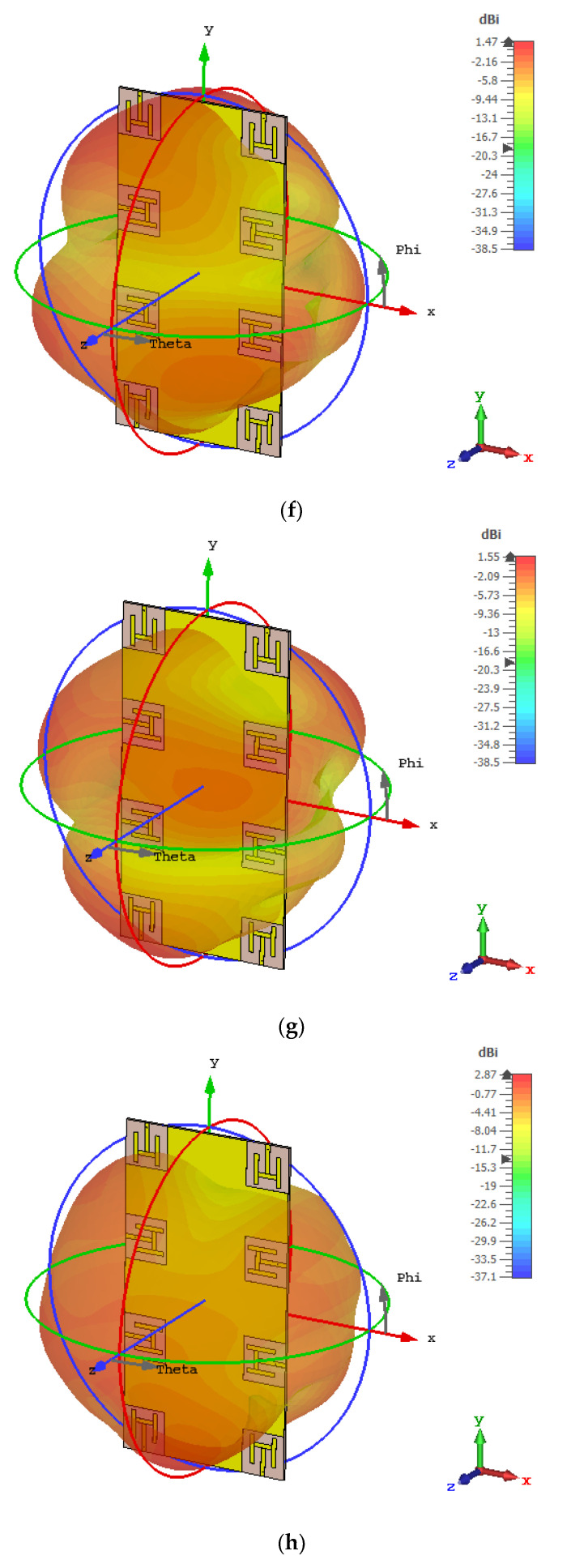
Radiation patterns at 3.5 GHz for (**a**) 1D Pattern Ant 2, (**b**) 1D Pattern Ant 4, (**c**) 1D Pattern Ant 6 (**d**) 1D Pattern Ant 8 (**e**) 3D Pattern Ant 2 (**f**) 3D Pattern Ant 4 (**g**) 3D Pattern Ant 6 (**h**) 3D Pattern Ant 8.

The radiation characteristics of the proposed system at 3.5 GHz is presented in Figure 6. Please note that for simplicity, only antennas at one-side of the board are considered. The far-field patterns for φ = 0° and φ = 90° planes are shown in the Figure 6. It is worthy to mention that the radiating elements are arranged on the board in such a manner so that the combined sum pattern of the system should be wideband and quasi-isotropic. Here, wideband means half power beam width (HPBW) should be wide, and isotropic means radiate in all directions over a sphere uniformly. Figure 6a illustrates the simulated and measured far-field pattern of antenna 2 for both *xz*- and *yz*-planes. For *xz*-plane, there is a null at +*y*-direction, while for *yz*-plane the main lobe is at θ = 90°. Similarly, the radiation pattern of antenna 4 for both planes are almost isotropic with maximum152 magnitude of the main lobe at θ = 30° and θ = −90°. Similar conclusions can be drawn for antenna 6 and antenna 8. It is worthy to mention that antenna 8 has similar patterns like antenna 2 because they are located at the corners, while antenna 6 and antenna 4 have similar characteristics as they are placed in the middle within the corner elements. The 3D radiation patterns at 3.5 GHz are shown in Figure 6e–h.

There is a minor alteration among experimental and simulated results in the far-field that may be due to material and fabrication tolerances. The peak Simulated gain obtained at central resonance frequency is 2.87 dBi while the measured gain obtained is 2.73 dBi. In summary, the simulated and experimental results are in very good agreement and the radiation patterns shown in Figure 6 cover complementary space regions, hence providing pattern diversity characteristics.

#### 3.2.3. MIMO Parameters

**Figure 7:**
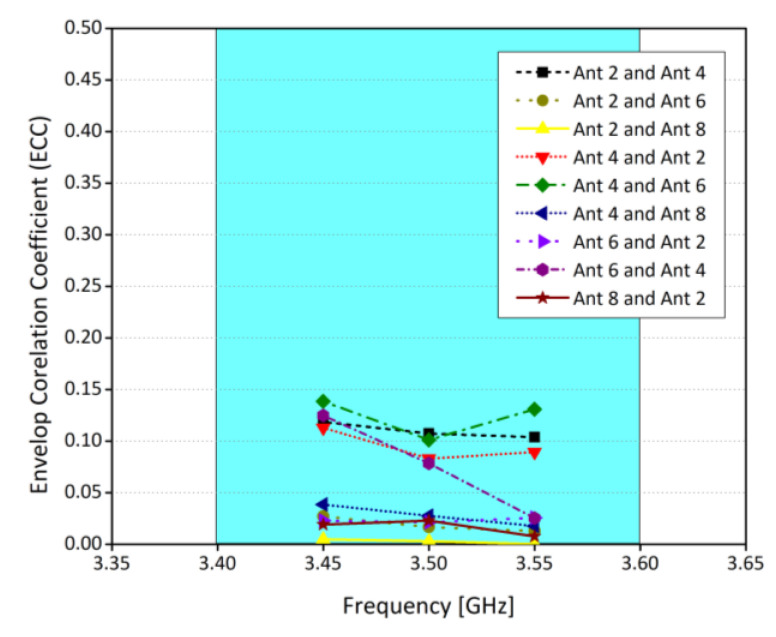
Envelope Correlation Coefficient (ECC) of proposed MIMO antenna.

**Figure 8:**
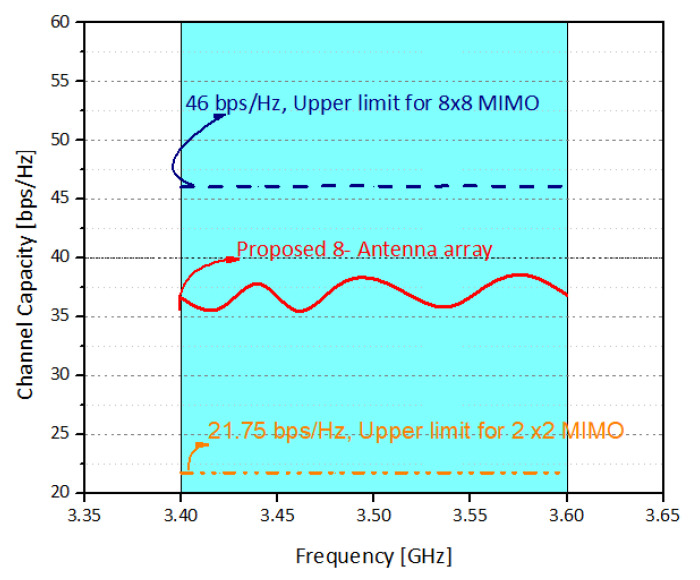
Channel capacity of proposed MIMO antenna.

**Figure 9:**
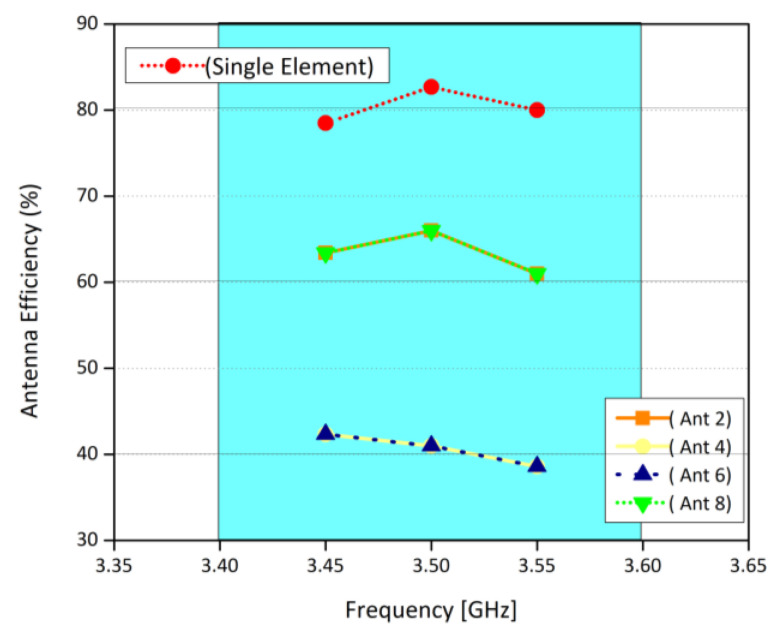
Antenna efficiency for single element and for MIMO system.

The ECC, MEG, and channel capacity are the key operation parameters of a MIMO system. They are used to evaluate the communication of the potential MIMO system. In MIMO systems, Envelope Correlation coefficient (ECC) plays an increasingly important role [23]. It defines how much the MIMO antenna elements are affecting each other. In other words, low ECC ensures better operation of MIMO systems, as the interference between the radiating elements is minimal. The ECC and MEG are calculated from the three-dimensional patterns of the electric field of the radiating elements within an array. It is worthy to note that, while calculating ECC and MEG, an assumption of uniform incident wave environment is made [11]. Figure 7 shows the ECC results between various antenna elements. It was observed that the ECC is well lower than 0.2 for all the cases considered, which is consistent with the international standards (ECC < 0.5) for 5G MIMO antenna systems. Similarly, MEG indicates the gain of the system within a multipath environment. In this work, the difference of MEGs between different radiating elements is well less than 1 dB for the whole operating range, as shown in Table 1. This means that the proposed eight-element array is suitable for practical MIMO applications. Figure 8 shows the channel capacity of the proposed MIMO system. Here, we have assumed uncorrelated transmitting antennas and identical independent channels with Rayleigh fading environment. From Figure 8, it is evident that the calculated channel capacities are about 38 bps/Hz within the desired frequency range, which is 2.9 times higher than the 4 × 4 MIMO system [24]. Similarly, the antenna efficiency of the single element is around 80%, while for the corner elements it is around 65% and for the elements in the middle, it is around 42%–40%, as shown in Figure 9. Please note that the efficiencies shown in the graph are total efficiencies and the radiation efficiencies is between 70 to 80% for corner antennas while for mid elements it’s in between 55 to 65%.

Based on the different analysis for various MIMO operation parameters, such as ECC and MEG which are well within the standards, it is believed that the proposed system is good for practical MIMO applications.

### 3.3. Hand Effect Analysis

In recent years, due to the advent of powerful smartphones, there is an increasing number of gaming applications. Nowadays, phones are not only used to communicate, instead they are used for multiple purposes. One such purpose is gaming. Moreover, in 5G technology, smartphones will be mostly used for data and less for voice [14,17,18]. Therefore, it is necessary to evaluate the operation of the proposed model for different scenarios, for instance the effect of hands. In this section, effects of two different modes of hand operations are studied, i.e., single-hand mode (SHM) and dual-hand mode (DHM), and key operation parameters of MIMO system, for example, ECC, scattering parameters, and efficiencies are analyzed under the influence of user hand(s). For the defined electric properties of customer’s hand, the target permittivity is 28 to 32 having effective conductivity is 0.7 to 0.9 S/m for hand phantom, but for conducting the user hand analysis in this study, the phantom hand model is inserted with a constant 29 permittivity and 0.8 S/m effective conductivity at centre frequency of 3.5 GHz [25,26].

**Figure 10:**
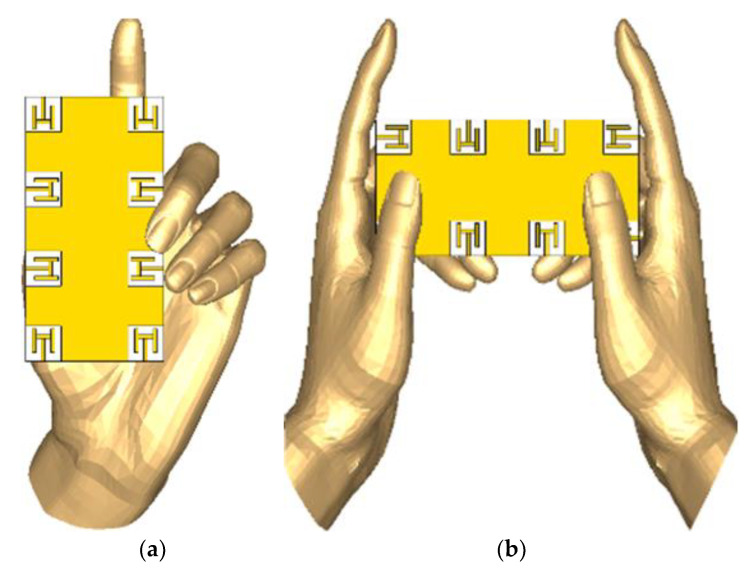
User hand analysis (**a**) single hand mode, (**b**) dual hand mode.

It is worthy to mention that the operation of the antenna is affected by the influence of the user hand in various different positions. For instance, positions of the fingers and/or gripping style, position, and placement of the palm of the hands, and palm of right- or left-hand. To understand and evaluate the effects of these and many other factors on the operation of the system, standard hand phantoms have been used in these studies. Both modes of operation, i.e., SHM and DHM, are illustrated in Figure 10. Here, the effective permittivity and effective conductivity is assumed to be 29 and 0.8 S/m, respectively. Please note that the antennas 2, 4, 6, and 8 do not have indirect contact with the fingers. 

**Figure 11:**
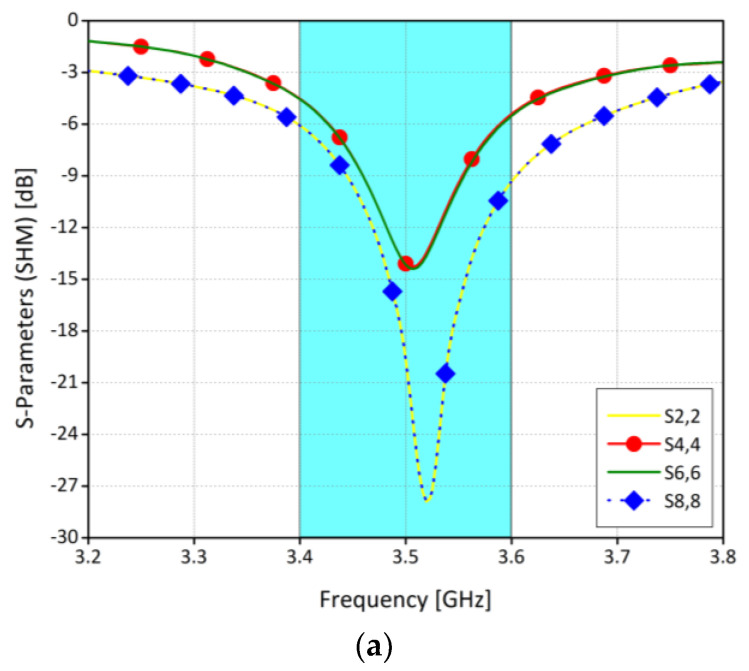

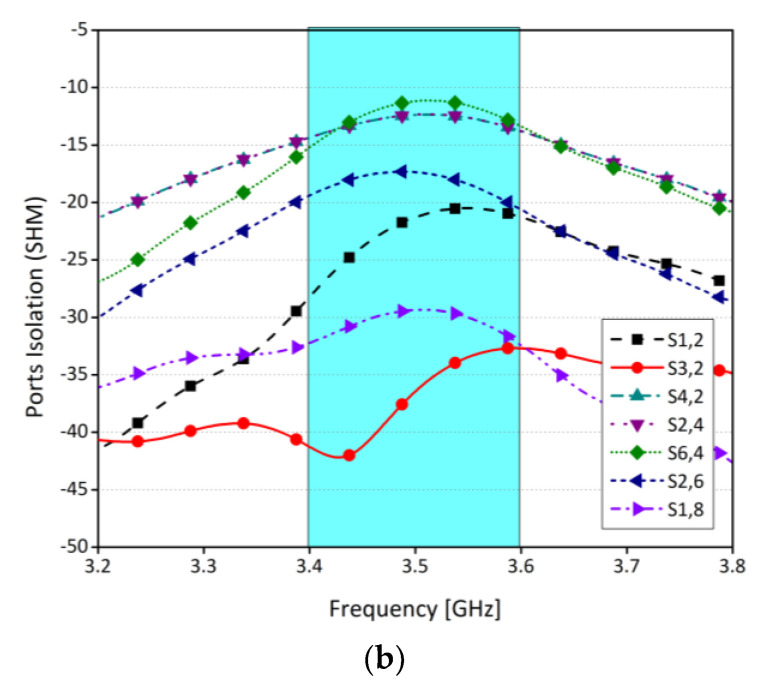
(**a**) Reflection coefficients for SHM. (**b**) Ports isolation for SHM.

**Figure 12:**
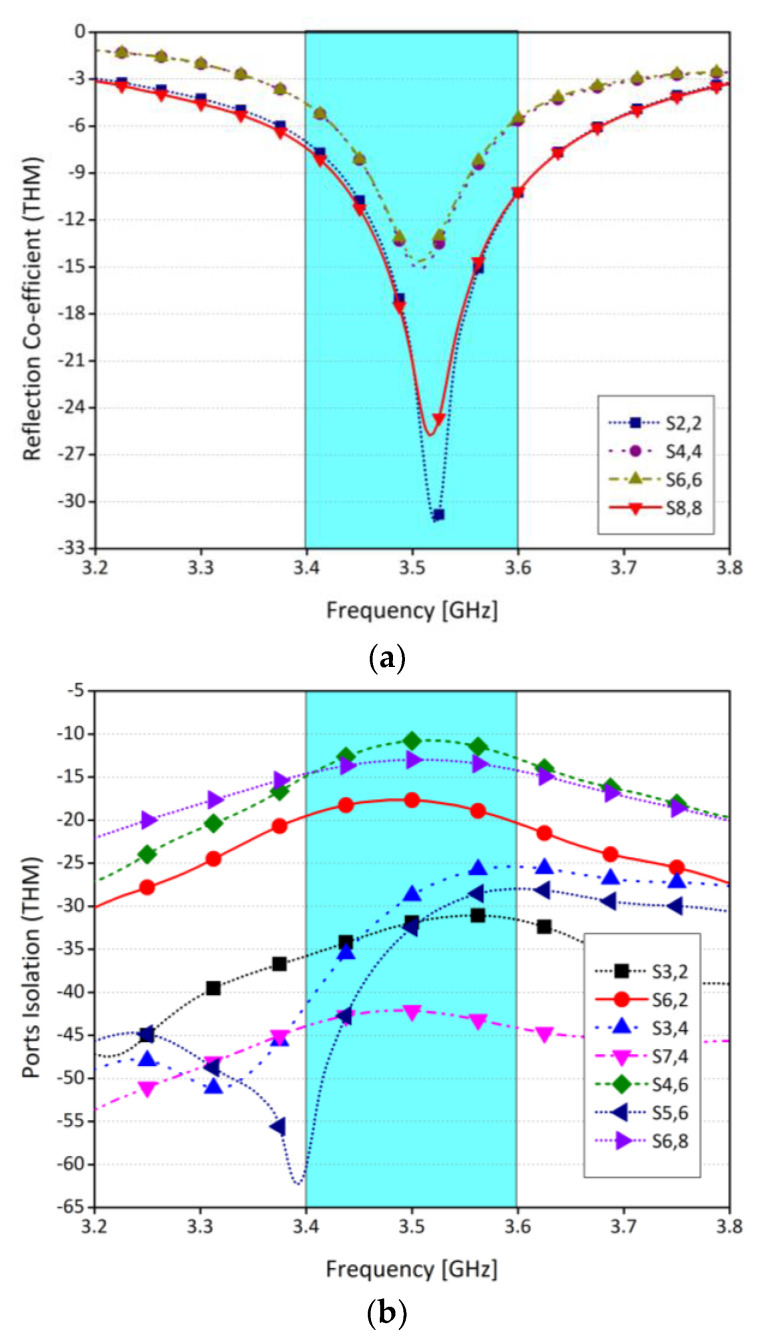
(**a**) Reflection coefficients for DHM, (**b**) ports isolation for DHM.

Figure 11 shows the scattering parameters of the system for SHM operation. From the figure, it is clear that, in SHM, a slight shift in the frequency from 3.5 GHz to 3.4 GHz is observed in the return loss, while isolation level is almost the same. The shift in the resonating frequency is due to the absorption of the energy within the user hand. Similarly, the scattering parameters for DHM operation are shown in Figure 12. It was observed that there is a very slight difference in with and without DHM operation. Also, the isolation level between the radiating elements was unchanged.

**Figure 13:**
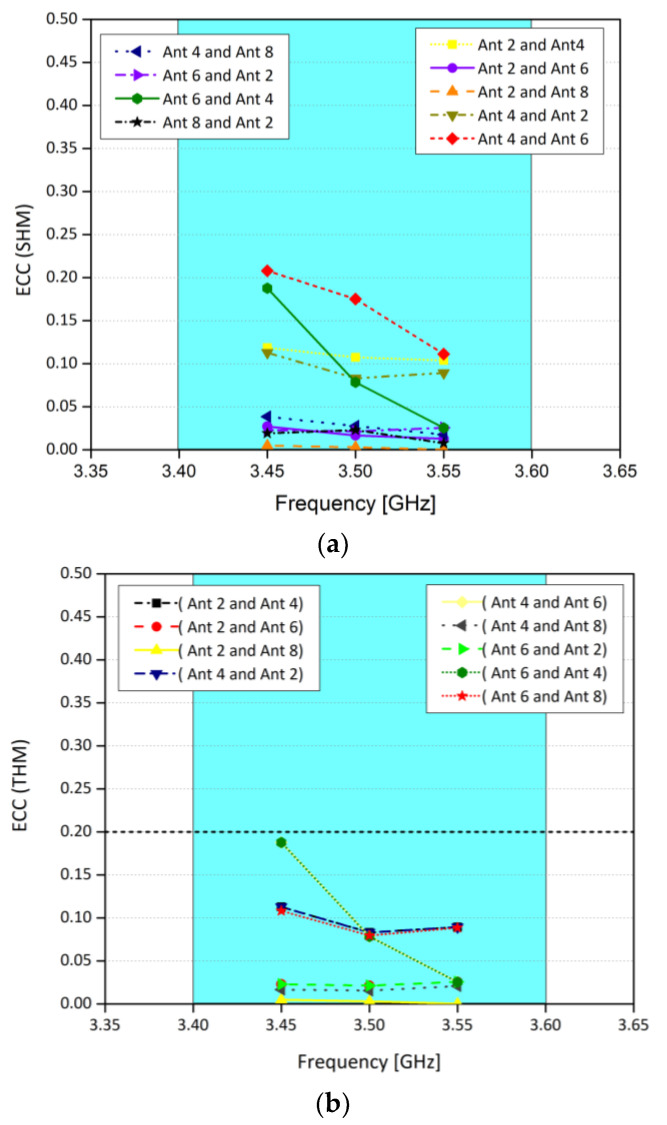
(**a**) ECC for SHM, and (**b**) ECC for DHM.

**Figure 14:**
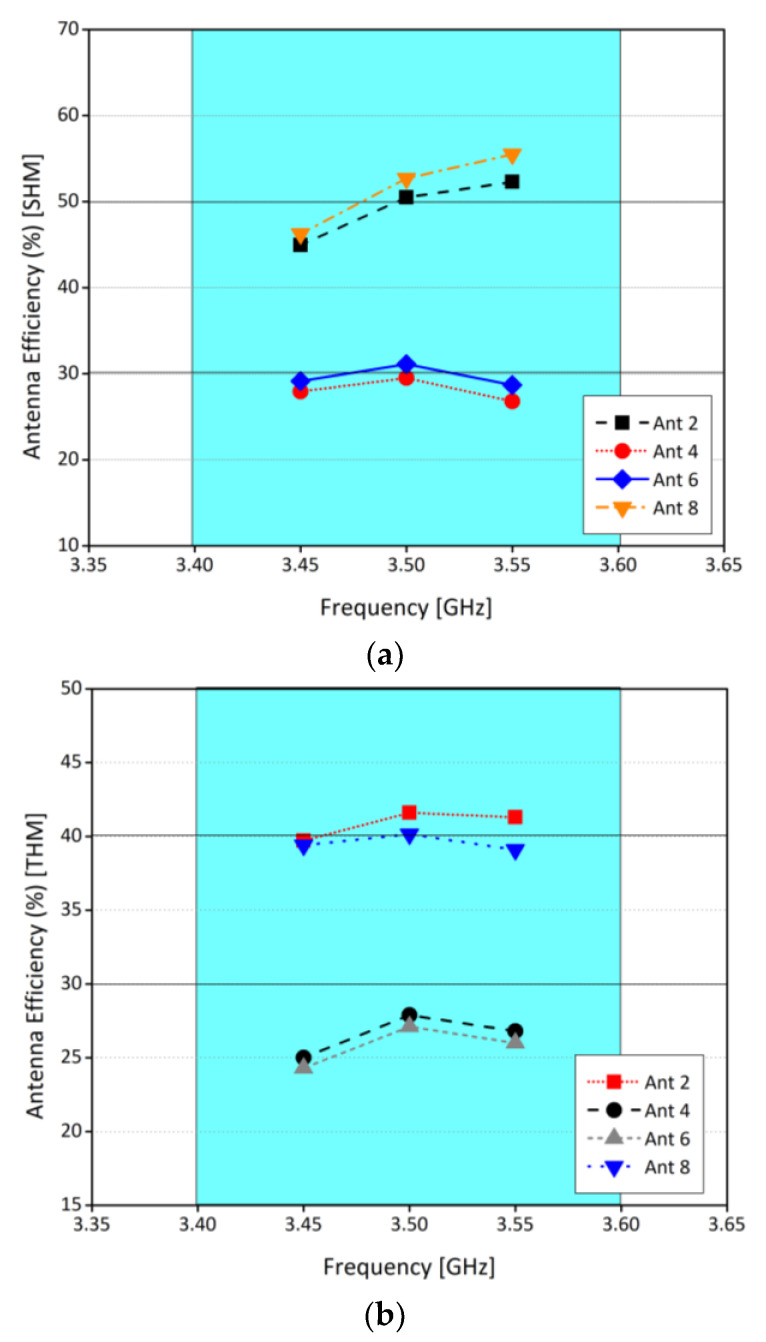
(**a**) Antenna efficiency for SHM (**b**) antenna efficiency for DHM.

To further investigate the effects of user hand operation, ECC for both modes of operation are considered, and it is found that ECC is less than 0.2 for any two radiating elements, as shown in Figure 13. Similarly, efficiencies for both modes of operation are illustrated in Figure 14. In SHM, within the operating frequency band, the efficiency of the antenna 2 and 8 is around 50%, while for antenna 4 and 6 it is around 30%. For DHM, for the same antenna sets, efficiencies are 40% and 28%, respectively. The decrease in the efficiency is due to the dielectric loading of the system, which in turn reduces the efficiency. Also, due to the interaction of the hand with the system leads to coupling as well. Moreover, a comparison of relative change in the efficiencies for free space mode, SHM and DHM is presented in Table 2. Please note that the efficiencies shown in Figure 14 are total efficiencies and the radiation efficiencies for SHM is between 60 to 70% for corner antennas while for mid elements it’s in between 45 to 55%. For DHM, radiation efficiency is between 45 to 55% for corner antennas while for mid elements it’s in between 35 to 40%.

Table 3 shows the comparison of our proposed antenna with published literature. In summary, in this work it is demonstrated that the proposed work is simple, low-cost, light-weight, and easy to fabricate and integrate with other RF devices and components. It is worthy to mention that based on different analysis, investigation, and studies, we are confident that the proposed model has potential to be a useful MIMO design for future 5G smart mobile terminals.

## 4. Conclusions

The main purpose of this work was to propose a simple antenna system that can fulfil 5G technology attributes in sub-6 GHz frequency band. An eight-element MIMO antenna system comprising an H-shaped monopole antenna was presented on an inexpensive FR4 substrate with 2.2 relative permittivity and 0.0002 loss tangent. The total size of the system is 150 × 75 × 0.8 mm^3^ and it was resonating at 3.5 GHz with a 200 MHz bandwidth. To allow other RF components and devices, the radiating elements and the ground plane are on the same side of the board. Four elements are etched on one side and the other four are placed symmetrically on the opposite side of the board. Different key operation parameters such as MEG, ECC, scattering parameters, channel capacity, and different studies such as user hand analysis are conducted to investigate the performance of the proposed system. Moreover, a prototype is fabricated, and it was found that the experimental results are in an excellent agreement with the simulated results. The isolation between any two radiating elements is less than 12 dB without using any decoupling structure, ECC is 0.2, and channel capacity is 38 bps/Hz. Also, it is observed that the proposed system is less affected by user hand operations. Therefore, it is reasonable to say that the proposed MIMO system can find its application in different future wireless technologies.

## Figures and Tables

**Table 1:** Calculated MEGs of MIMO Antenna.

Frequency	3.50 GHz	3.45 GHz
MEG1	−4.659	−4.932
MEG2	−5.012	−5.321
MEG3	−5.797	−5.881
MEG4	−4.987	−4.965
MEG5	−5.176	−5.581
MEG6	−5.413	−5.678
MEG7	−5.011	−5.317
MEG8	−5.230	−5.310

**Table 2:** Percentage Decrease in S.H.M and D.H.M mode w.r.t Free Space.

Antenna Operational Mode		Efficiency in 3.5 GHz Band (%)	Percentage Decrease in Efficiency w.r.t Free Space Mode (%)
Free Space	Ant 2 & Ant 8	64	Not Applicable
	Ant 4 & Ant 6	38	
SHM	Ant 2 & Ant 8	50	21.8
	Ant 4 & Ant 6	30	21
DHM	Ant 2 & Ant 8	40	37.5
	Ant 4 & Ant 6	28	26.3

**Table 3:** Proposed Antenna Comparison with Published work.

Refs.	Frequency (GHz)	Elements	Elements Size	Efficiency (%)	Board Size	Channel Capacity	Isolation (dB)	Gain (dBi)	ECC
[1]	3.4–3.6 (−10 dB)	8	14 × 6	62–76	150 × 75	38.5	<−12	N/A	<0.05
[3]	3.4–3.6 (−10 dB)	8	14.2 × 9.4	>40	145 × 70	N/A	−16	2	<0.2
[4]	3.45–3.55 (−6 dB)	4	25 × 13	40–50	120 × 73	15	<−15	1.9	<0.31
[6]	3.4–3.6 (−10 dB)	6	8.5 × 3	50–60	136 × 68	31.25	<−13	4.8	<0.15
[8]	2.5–3.6 (−10 dB)	8	7 × 6	45–65	150 × 70	34.25	<−15	2.3	<0.2
[25]	3.4–3.6 (−10 dB)	8	21.5 × 3	62–76	150 × 80	40.8	<17.5	N/A	<0.05
[26]	3.3–3.7 (−6 dB)	8	4.6 × 5.6	50–70	136 × 68	38.1	−15	4	<0.1
Proposed	3.4–3.6 (−6 dB)	8	12.5–18.5	42–65	150 × 70	38	<−12	2.87	<0.2

## Data Availability

All date have been included within the manuscript.
